# The higher exercise intensity and the presence of allele I of ACE gene elicit a higher post-exercise blood pressure reduction and nitric oxide release in elderly women: an experimental study

**DOI:** 10.1186/1471-2261-11-71

**Published:** 2011-12-02

**Authors:** Hugo AP Santana, Sérgio R Moreira, Willson B Neto, Carla B Silva, Marcelo M Sales, Vanessa N Oliveira, Ricardo Y Asano, Foued S Espíndola, Otávio T Nóbrega, Carmen SG Campbell, Herbert G Simões

**Affiliations:** 1 Programa de Pós-graduação em Educação Física e Saúde, Universidade Católica de Brasília - UCB, Brasília-DF, Brazil; 2 Centro Universitário do Planalto de Araxá - UNIARAXÀ; Araxá-MG, Brazil; 3 Universidade Federal do Vale do São Francisco -UNIVASF- Petrolina-PE, Brazil; 4 Faculdade de Educação Física do Centro Anhanguera Educacional, Taguatinga-DF, Brazil; 5 Faculdade de Educação Física do Centro Universitário - UNIRG -, Gurupi-TO, Brazil; 6 Instituto de Genética e Bioquímica da Universidade Federal de Uberlândia - UFU, Uberlândia-MG, Brazil; 7 Programa de Pós-Graduação em Ciências Médicas e Programa de Pós -graduação em Ciências da Saúde, Universidade de Brasília - UnB, Brasília-DF, Brazil

## Abstract

**Background:**

The absence of the I allele of the angiotensin converting enzyme (ACE) gene has been associated with higher levels of circulating ACE, lower nitric oxide (NO) release and hypertension. The purposes of this study were to analyze the post-exercise salivary nitrite (NO_2_^-^) and blood pressure (BP) responses to different exercise intensities in elderly women divided according to their ACE genotype.

**Methods:**

Participants (n = 30; II/ID = 20 and DD = 10) underwent three experimental sessions: incremental test - IT (15 watts workload increase/3 min) until exhaustion; 20 min exercise 90% anaerobic threshold (90% AT); and 20 min control session without exercise. Volunteers had their BP and NO_2_^- ^measured before and after experimental sessions.

**Results:**

Despite both intensities showed protective effect on preventing the increase of BP during post-exercise recovery compared to control, post-exercise hypotension and increased NO_2_^- ^release was observed only for carriers of the I allele (p < 0.05).

**Conclusion:**

Genotypes of the ACE gene may exert a role in post-exercise NO release and BP response.

## Background

The systemic arterial hypertension (SAH) has committed about two thirds of elderly population in several countries [1,2]. Being considered a cardiovascular risk factor [3] that may be associated with endothelial dysfunction and thus with a low endothelial dependent vasodilatation [4,5].

The treatment of SAH includes pharmacological therapy and lifestyle changes, such as physical activity enrollment and nutritional habits re-education [6]. Among cardiovascular benefits of exercise, the post-exercise blood pressure reduction has been considered an important tool for blood pressure (BP) control [7-15]. The protective effect of exercise in lowering blood pressure may be mainly due vasodilatation substances induced vascular resistance reduction after exercise [9].

During physical exercise the increased blood flux lead to mechanical vessel stress and thus an endothelial NO release [16]. The NO is involved with vascular tonus regulation [17] and its release has been associated to post-exercise BP reduction (e.g. post-exercise hypotension - PEH), which may even be influenced by genetic characteristics [18].

Hypertensive people would benefit from PEH as a non-pharmacological adjunct to the SAH treatment. However, Hagberg et al. [19] highlighted that approximately 25% of the hypertensive individuals do not present PEH, what could be partially explained by genetic variations.

Studies about the insertion/deletion (I/D) polymorphism of angiotensin converting enzyme (ACE) and its associations to functional, metabolic and cardiovascular phenotypes have been documented [20-22], including SAH [10,23]. The absence of I allele of ACE gene (e.g. individuals D/D) has been associated with higher levels of circulating ACE [24-26] leading to an increased angiotensin II (ANG II) concentration, reduced bioavailability of bradykinin and thus to a lower NO release [27,28].

We hypothesized that in respect to ACE gene, the D/D carriers would present lower NO release during exercise and thus both lower vasodilatation and reduced post-exercise blood pressure reduction than those with the I allele. Furthermore, once the endothelial release of NO is dependent on both metabolic and mechanical stress (shear stress) [16], it was also hypothesized that aerobic exercise of a greater intensity would result in a higher NO release and thus to elicit a greater post-exercise BP reduction in elderly hypertensive women.

Therefore, the purposes of this study were to analyze the post-exercise NO and blood pressure responses to different exercise intensities in elderly women with or without the I allele of ACE gene, as well as to investigate if the NO release and post-exercise hypotension in this population would be influenced by of the I/D polymorphism of ACE gene. Due to factors such as age and gender affect blood pressure responses, this report poses a contribution by standardizing these variables in our sample.

## Methods

### Participants

In the initial phase of this study 268 elderly women (≤ 60 years-old) clinically diagnosed with hypertension were genotyped for the I/D polymorphism of ACE gene. The diagnosis of hypertension in this sample was performed in 2005 and confirmed in 2006/2007 at the medical department of the University according to IV Brazilian Hypertension Guidelines (2006) [29] following procedures previously described by Moraes et al. (2008) [30]. From these initial volunteers, thirty elderly women (70.5 ± 6.0 years, 60.4 ± 8.5 kg, 153.3 ± 6.3 cm and 25.7 ± 3.0 kg/m^2^) diagnosed with mild hypertension and whose pharmacotherapy consisted only on diuretics as hydrochlorothiazide and indapamide (not interfering with the RAS) were selected to enrolled in a local program for SAH treatment which included regular physical activity and recommendation of a balanced food intake.

After recruitment, participants were allocated into two different groups according to the presence (II and ID) or absence (DD) of 287 pairs of base. Participants of both groups (II/ID and DD) randomly underwent to three experimental test sessions on non-consecutive days, inter a spread with at least 48-hours apart. After giving a written consent, each volunteer was first submitted to a resting electrocardiogram, and exercise tests were performed under cardiologist's supervision. The study was approved by the local ethical committee (process nº CEP/UCB 63/2008).

### General Procedures

All 268 women had a blood collection for the determination of their I/D polymorphism of ACE gene. After that, 30 volunteers were selected according to their availability to underwent to testing protocol, their ACE genotype (individuals with and without the I allele of ACE) as well as considering the exclusion criteria (e.g. use of medicines that would interfere on RAS). Before each experimental session, all volunteers remained on resting for 20 minutes, and blood pressure (BP) was measured every five minutes, being the average considered the resting BP. Experimental design consisted in an incremental test session, one session at 90% of anaerobic threshold and another session without exercise (control session). After all sessions, the volunteers remained in the laboratory for recovery during one hour. In this time span, the post-exercise BP was measured every 15 min, and the mean of these measures was considered. NO was inferred from the measurement of nitrite (NO_2_^-^) in saliva [31-34]. For this analysis, saliva was collected during resting before exercise (or control) in all testing days (PRE), immediately after exercise (IAE) and concomitantly to BP measurements throughout the recovery period. The saliva was collected through a cotton swab.

### ACE Genotyping

Total DNA was isolated from peripheral blood according to standard procedures. The insertion(I)/deletion(D) polymorphism in the human ACE gene (rs4646994) was determined by inspection of the electrophoretic profile of polymerase chain-reaction (PCR) products, and performed as described by Marre et al. with modifications [34]. Either the 490 bp (I allele) or the 190 bp (D allele) products were amplified using primers: 5′-CTGCAGACCACTCCCATCCTTTCT-3′ and 5′-GATGTGGCCATCACATTCGTCAGAT-3′, which flank the polymorphic site. Reaction tubes contained 100 ng DNA, 10 mmol/L Tris-HClpH8.3,75 mmol/L KCl, 3.5 mmol/L MgCl2, 0,2 mmol/L dNTP, 20 pmol of each primer, 0.5 μg of purified chicken albumin and 1 U of Taq DNA polymerase (Phoneutria^®^, Minas Gerais, Brazil) in a final volume of 25 μL. After 1 min of hot start at 80 °C and an initial denaturation for 2 min at 94 °C, the amplifications were done for 30 cycles of 40 s at 94 °C, 45 s at 64 °C and 50 s at 72 °C followed by a final 5 min extension at 72 °C. Inspection of DD subjects was carried out using oligonucleotides (5′-TGGGACCACAGCGCCCGCCACTAC-3′ and 5′-TCGCCAGCCCTCCCATGCCCATAA-3′) specific to amplify a 335 bp fragment of the insertion sequence. In brief, DNA was amplified for 30 cycles with denaturation at 92 °C for 40 s, annealing at 63 °C for 40 s, and extension at 72 °C for 40 s. All PCR products were separated by electrophoresis on 2% agarose gels containing ethidium bromide at 50 μg/ml, visualized by using CCD camera (Vilber Lourmat^®^, Eberhardzell, Deutschland), examined using the gel analysis software enclosed (Photo Capt 1D), and confirmed by visual inspection.

### Incremental Test and Anaerobic Threshold (AT) determination

The volunteers performed a maximal incremental test (IT) in cycle ergometer (Lode Excalibur, Netherlands) that consisted in 1-min warm-up at 0 watts followed by a pace of linear, incremental gradient in 15 watts every 3 minutes stage. The test was terminated due volitional exhaustion, incapacity of maintaining 60 rpm or if any cardiovascular-related risk was detected by cardiologist. In each stage of IT, a blood sample was withdrawn from earlobe to assess blood lactate concentration [Lac]. Measures of rate of perceived exertion (RPE) as well as ventilation (VE), oxygen uptake (VO_2_) and carbon dioxide production (VCO_2_) (Cortex Metamax, Leipzig, Germany) were performed at the end of each stage.

The AT intensity was determined by assessing the ventilatory threshold (disproportional increase in the ventilatory equivalent for oxygen (VE/VO_2_) in relation to ventilatory equivalent for dioxide carbon (VE/VCO_2_) and the [Lac] turnpoint (workload corresponding to deflection point where the concentration of blood lactate increased disproportionally). The AT was considered the mean workload (watts) between ventilatory and lactate thresholds.

### Sub-maximal constant load exercise test

Participants underwent to a constant load exercise test at intensity corresponding to 90% AT. During the 20 min exercise at 90% AT, expired gases were measured continuously and the RPE were asked at the 10^th ^and at the end of exercise. This intensity was chosen due to benefits on blood pressure and cognitive performance observed in other studies in elderly individuals [11,35].

### Control Session

During control session the volunteers remained in resting for 20 min instead of exercising. However, all measurements were the same as those performed during a constant load exercise session.

## Measurements

### Blood Lactate and Gases Analyses

In the incremental test on cycle ergometer, blood samples were drawn and expired gases were collected during the last 20 seconds of each incremental stage. The blood was collected in microcapillary heparinized tubes and deposited in microtubes containing 50 μL of sodium fluoride (1%) for [Lac] measurements through an electrochemical analyzer (YSI 2700, YSI, Inc., Yellow Springs, OH, USA). Expired gases were collected breath by breath (Cortex Metamax, Leipzig, Germany). For the 90% AT and CONT groups, blood collection occurred at the 10^th ^and 20^th ^minutes; however gas measurement occurred during the whole 20 min span.

### Heart Rate, Rate of Perceived Exertion and Blood Pressure

During the all sessions, the heart rate (HR) (Polar s810i^®^, Kempelle, Finland), a 15 point RPE scale [36] and blood pressure (BP) (Microlife BP3AC1-1, Berneck, Switzerland) were determined. All resting and post-exercise BP measurements were made according to the procedures of JNC 7 [2] on the participant's left arm while they were seated with their feet on the ground and arm resting comfortably at the level of the heart.

### NO Metabolic Measurement in saliva

Saliva was collected with a cotton swab (Salivette Sarstedt^®^, Nümbrecht, Germany) which was chewed for one minute. Then it was centrifuged according to the manufacture instructions and stored in -20°C for latter analysis. Dosage of nitrite (NO_2_^-^) a NO metabolite [31-33] was done through the Griess' colorimetric method^22^. Briefly, N-(1-naphthyl)-ethylenediamine (NED) (Sigma^®^- Aldrich, St. Louis, USA) was prepared at 0.1%, whereas sulfanilamide (Sigma^®^) at 1%, both with phosphoric acid at 2.5% as diluent. Saliva (50 μL) and the Griess' reagent (50 μL) were mixed and placed in microplates. Absorbance was measured at 450 nm, in Versamax tunable^® ^(Molecular Devices, Sunnyvale, California, USA), and sodium nitrite (NaNO_2_^-^) was used as a standard. The data were analyzed in the Microplate^® ^software. Saliva samples of only 28 (II/ID - n = 18, DD - n = 10) elderly women were processed due technical problem in collecting procedures of two volunteers that unable to run the analyses.

### Statistical Analyses

An exploratory analysis was used to verify data normality and then descriptive statistics were performed. Data are presented as means (± standard deviation) for BP and means (± standard error of mean) for NO_2_^-^. In addition, the delta variations (absolute variation from rest to post-exercise values) were calculated for comparison. Student's t-test and One-Way ANOVA for repeated measures were used to compare experimental sessions. The Tukey test was adopted as a *post hoc *to identify differences. The level of significance was set at p ≤ 0.05.

## Results

The general characteristics of the volunteers and power output, aerobic fitness, heart rate, metabolic variables and RPE results of IT and 90%AT according to the genotypes of the investigated groups are presented on table 1.

**Table 1:** General characteristics and descriptive data of the studied groups of carriers or non-carriers of "I" allele of ACE gene (n = 30).

	I/D-I/I	D/D	*p*
N	10/10	10	-
Age (years)	70.4 ± 6.2	70.6 ± 5.8	0.93
Weight (kg)	60.6 ± 8.4	60.0 ± 9.1	0.86
Height (cm)	153.1 ± 7.1	153.7 ± 4.7	0.82
BMI (kg·m^-2^)	25.9 ± 3.0	25.4 ± 3.2	0.70
***Metabolic and Hemodynamic Variables***			
Glycaemia (mg·dL^-1^)	86.9 ± 13.1	79.6 ± 17.1	0.21
SBP IT rest (mm·Hg^-1^)	129.0 ± 17.0	125.0 ± 14.0	0.47
DBP IT rest (mm·Hg^-1^)	77.0 ± 8.0	77.0 ± 7.0	0.87
NO_2_^- ^rest (μM)	294.4 ± 176.5	278.2 ± 159.0	0.81
***Performance data of IT and 90%AT***			
Watts peak IT (Watts)	62.3 ± 21.4	57.0 ± 19.7	0.52
Watts at AT (Watts)	38.4 ± 14.4	39.6 ± 14.8	0.83
Watts at 90%AT (Watts)	34.5 ± 13.0	35.6 ± 13.3	0.83
VO_2_peak IT (mL·kg^-1^·min^-1^)	20.5 ± 4.3	20.1 ± 3.0	0.78
VO_2 _at 90%AT(mL·kg^-1^·min^-1^)	14.8 ± 2.3	15.8 ± 3.3	0.37
HRpeak IT (bpm)	148.0 ± 21.0	142.7 ± 19.6	0.51
HR at 90%AT (bpm)	119.5 ± 15.8	116.8 ± 17.6	0.68
[Lac] peak (mM)	4.8 ± 1.9	4.4 ± 1.4	0.54
[Lac] 90%AT (mM)	3.0 ± 1.1	3.3 ± 1.2	0.42
RPE peak IT (Borg)	17.6 ± 1.5	18.1 ± 1.6	0.41
RPE at 90%LA (Borg)	13.2 ± 2.0	13.8 ± 1.2	0.42
Diuretics Medication (% of volunteers)	20.0	20.0	-

I/D-I/I and D/D - angiotensin converting enzyme (ACE) genotype; BMI - body mass index, SBP - systolic blood pressure, DBP - diastolic blood pressure, NO_2_^- ^- nitrite concentration, VO2peak: peak oxygen consumption reached in IT; VO2: oxygen consumption; HRpeak: peak heart rate reached in IT; HR: heart rate; [Lac] peak: peak lactate concentration reached in IT; [Lac]: lactate concentration; RPE peak: peak rate of perceived exertion reached in IT; RPE: rate of perceived exertion.

The systolic blood pressure (SBP), diastolic blood pressure (DBP) and mean arterial pressure (MAP) are presented on table 2 as related to the studied ACE genotype groups. Since the data did not present any significant differences for the resting values (P > 0.05), the delta variation results (post-exercise values minus resting values) was also used to analyze variation among sessions.

**Table 2:** Blood Pressure results at pre and post-sessions as well as the post-exercise delta variation in relation to pre-exercise resting for the ID/II and DD groups.

			IT			90%AT			CONT	
		Rest	Mean 1 h	Δ variation	Rest	Mean 1 h	Δ variation	Rest	Mean 1 h	Δ variation
**SBP**	**ID/II**	129.1 ± 16.9	121.7 ± 14.7*	-7.4 ± 8.4*^‡#^*	123.3 ± 12.8	119.1 ± 12.8*	-4.2 ± 6.0*^‡#^*	122.0 ± 17.6	125.1 ± 16.3*^†^*	3.1 ± 7.1
**(mmHg)**	**DD**	124.5 ± 13.9	122.5 ± 13.7	- 2.0 ± 3.6	117.7 ± 11.4	119.7 ± 14.8	2.0 ± 8.7	118.4 ± 7.4	121.7 ± 8.3	3.3 ± 5.7
**DBP**	**ID/II**	77.0 ± 8.4	77.3 ± 8.6	0.3 ± 6.8	75.3 ± 7.1	75.7 ± 5.6	0.4 ± 3.7	73.8 ± 7.8	76.6 ± 7.4*	2.8 ± 2.4
**(mmHg)**	**DD**	76.5 ± 7.1	77.8 ± 7.8	1.3 ± 6.3	71.9 ± 4.1	74.6 ± 6.9	2.7 ± 5.2	73.0 ± 6.7	76.0 ± 7.3*^†^*	3.0 ± 4.4
**MAP**	**ID/II**	94.3 ± 10.3	92.1 ± 9.9	-2.2 ± 5.9*^‡^*	91.3 ± 8.5	90.2 ± 7.2	-1.1 ± 3.9*^‡^*	89.9 ± 10.2	92.8 ± 9.8*	2.9 ± 3.1
**(mmHg)**	**DD**	92.5 ± 8.9	92.7 ± 9.1	0.2 ± 4.7	87.2 ± 6.3	89.6 ± 9.2	2.4 ± 6.1	88.1 ± 6.6	91.2 ± 7.3*	3.1 ± 4.4

IT - Incremental Test session, 90%AT - 90% of anaerobic threshold session, CONT - control session. *p < 0.05 to rest in the same session; †p = 0.06 to rest in the same session; ‡p < 0.05 to Cont in the same group, #p < 0.05 to D/D in the situation.

During the post-exercise recovery from IT the SBP values (table 2) were significantly lower than pre-exercise resting for the II/ID group both for the IT and 90%AT sessions. These variations when analyzed in delta were significantly lower (p < 0.05) to control session and to D/D groups in the same circumstances. The DBP and MAP on the control sessions present differences (p < 0.05) or at least a trend to it (p = 0.06) for the D/D group for DBP, from the rest to 1 h Mean recovery time. The delta variation of MAP presented significant negative values for the IT and 90%AT sessions being significant different (p < 0.05) to the control delta variation.

**Figure 1:**
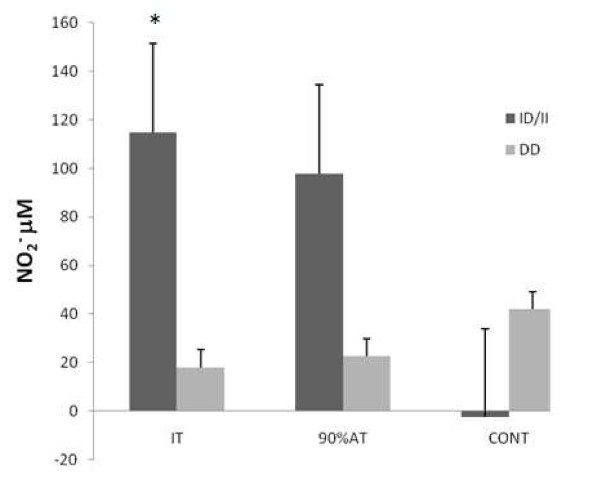
**Mean (± SEM) delta variation of NO_2_^- ^(nitrite) for IT (Incremental test), 90% AT (90% of anaerobic threshold) and CONT (control) sessions performed by ID/II (n = 18) and DD (n = 10) genotypes of ACE gene**. * p < 0.05 in relation to D/D group on IT session.

The NO_2_^- ^(table 3) presented a significantly higher (P < 0.01) values immediately after experimental session at IT (IAE) and a trend to be higher (P = 0.08) at the 90%AT session too (Figure 1) in comparison to resting on the group that presented the I allele of ACE gene.

**Table 3:** Nitrite (NO_2_^-^) concentrations in rest and immediately after experimental session in groups separated by ACE genotypes (ID/II - n = 18; DD - n = 10).

		TI		90%AT		CONT	
		Rest	IAE	Rest	IAE	Rest	IAE
**NO_2_^-^(μM)**	**ID/II**	286.6 ± 29.4	401.3 ± 52.8*	239.8 ± 34.6	337.5 ± 63.8^†^	295.5 ± 39.4	292.9 ± 44.9
	**DD**	264.9 ± 70.5	282.9 ± 63.8	318.5 ± 65.0	341.2 ± 93.6	251.1 ± 44.8	293.0 ± 94.2

IT - incremental test session, 90% AT - session at 90% of anaerobic threshold, CONT - control session, NO_2_^- ^- nitrite, IAE - immediately after experimental session. *p < 0.01 in relation to rest in the same session; ^†^p = 0.08 in relation to rest in the same session.

The delta variation of NO_2_^- ^presented significant differences (p < 0.05) with a higher NO_2_^- ^release after IT for the II/ID group when compared to DD group as shown in Figure 1.

## Discussion

This study analyzed the BP responses after different exercise intensities and the NO_2_^- ^release as related to ACE genotypes in elderly women. The main findings were that both the exercise intensity and the presence of I allele of ACE gene may interfere on NO_2_^- ^liberation and post-exercise hypotension (PEH) occurrence in hypertensive elderly women. PEH occurred for the SBP among carriers of the I allele only. Also, those I carriers presented lowered post-exercise blood pressure levels in relation to the DD group. Despite the intensity of the exercise sessions, both intensities were effective for lowering the resting values of BP whereas these values increased in the control session, without exercise.

The fact that only elderly women that had the I allele presented PEH of SBP after both exercise sessions (table 2) is probably due to the fact that D/D homozygote presents almost twice as higher the angiotensin converting enzyme activity when compared to the insertion homozygote [24,25]. The role of ACE is mainly to convert angiotensin I to angiotensin II. This last action are related to an increased sympathetic tone induced arteriolar constriction and release of aldosterone from supra-renal cortex [22,37,38] which, in turn acts in kidneys, leading to potassium excretion, salt reabsorption and water retention. All those effects may elevate the BP, and in theory would interfere in the post-exercise BP achievement.

Additionally, the fact that DD individuals present a higher circulating level of ACE [25] may lead to a higher activity of ANG II that may further blind the vasodilation induced by bradykinin [37] and thus influence the BP values [22]. This mechanism causes a negative impairment on the endothelial dependent dilation, once it reduces the bioavailability of NO [16] what, in turn, would be the reason of non significant post-exercise blood pressure reduction for the D/D group in any experimental session of the present study.

The findings of PEH of SBP in present study, for the group that presented the I allele, were similar to others studies. Pescatello et al. (2007) [10] analyzed the BP response after high and low calcium ingestion and after two sessions of low and moderate exercise intensity, and verified in the intensity corresponding to 60% VO_2 max_, the I allele carriers that had low calcium ingestion presented PEH of higher magnitude for the SBP. However, for the DBP no ACE genotypes interactions were found. For the present study, besides the main effect on SBP, the exercise also presented a protective effect on post-exercise DBP and MAP despite the genetic profile compared to control (table 2).

Blanchard et al. (2006) [39] verified for 14 h the ambulatory BP in adult men at the same intensities studied by Pescatello et al. (2007) [10] and the results were contradictory to ours, presenting increases in the mean of 14 h in the SBP and DBP for all experimental sessions (60% VO_2_max, 40% do VO_2_max and control) despite the genetic combinations of RAS, however the exercise sessions had benefits when compared to control. Moreover, they found benefits of post exercise SBP 14 h after light exercise session (40% VO_2_max) with lower values for the DD homozygote but not for the I allele group. These distinct results may be either related to gender differences, because some studies suggest that associations between BP and genotype DD of ACE gene are only significant in men showing some effect on the BP [40,41], or to age differences, because in present study the sample was composed by elderly people, that presents different endothelial responses when compared to youngsters [42].

The BP increase in the control session can be partly explained from the waiting time (60 minutes) until the end of data collection procedures, that may have produced some degree of distress that may have contributed to the augmentation observed. Zimmerman & Frohlich [43] related that acutely, stress episodes have been shown to increase blood pressure by increasing cardiac output and heart rate but without affecting peripheral resistance. In addition, even moderate stress has been found to increase levels of catecholamines, cortisol, vasopressin, endorphins and aldosterone, which may in part explain the increase in blood pressure. This may not have occurred in the experimental session (90% AT) due to the protective effect of exercise in situations of acute stress, as observed by MacDonald et al. [44].

Furthermore, the increase in BP even with the non-significant of NO_2_^- ^(p > 0.05) augmentation in the DD group may be due to overlap of complementary input signals, with a probable prevalence of humoral and neural mechanisms in blood pressure control. Studies [45-47] have reported that individuals carrying the DD genotype have higher levels and activity of the angiotensin converting enzyme (ACE), which therefore could result in a greater increase in blood pressure by increasing the conversion of angiotensin I to II, causing vasoconstriction and also enhancing water and sodium reabsorption by the kidneys, increasing blood volume and blood pressure.

The trends of a higher exercise intensity to be more effective on inducing PEH (table 2) are in accordance to former results in our laboratory, but on individuals with type-2 diabetes [11]. The possible role of exercise intensity on the present study was demonstrated for the results of NO release as well (table 3). The NO_2_^- ^results of this study reinforce the important role of the NO on reducing BP, as already demonstrated by other authors [2,42,48].

Nevertheless, Lauer et al. (2008) [42] showed that elderly, when compared to youngsters, has endothelial dysfunction being harder in increase plasmatic NO_2_^- ^in response to exercise. However, it was interesting to demonstrate in our research that in elderly population the ability to increase NO_2_^- ^may be intensity-dependent, and may be associated to genetic characteristics with the DD group not presenting significant changes in NO_2_^- ^and these findings together are the main contribution of the present study.

Tanriverdi et al. (2005) [49] verified that flux mediated dilation response in athletes presenting that II, was higher than ID and DD genotypes, being the homozygote D with the worst response to flux mediated dilation, what corroborates with our results (no PEH and lower NO_2_^- ^release for the DD group).

The increased liberation of the NO_2_^- ^after exercise sessions may occurs due to shear stress in the blood vessels what stimulates the endothelial NO formation [50,51]. The fact that NO_2_^- ^being significantly higher only after the IT session, on the I/D - I/I group, may also be due to exercise at higher intensity to promote a more significant shear stress [52], even for elderly that may be predispose to endothelial dysfunction and low NO release [42,53,54].

The study limitations were not measuring the endothelial nitric oxide synthase (eNOS) what could represent the endothelial dependent activity and, consequently, a possible PEH. However, some authors [31-33] verified that the NO_2_^- ^in saliva predicts the plasmatic NO_2_^- ^concentration that is one of better eNOS activity indexes [55]. Another limitation of this study was the lack of ACE measurement in the elderly participants; however some authors [24,25] observed higher values of this activity with homozygote D when compared to other ACE genotypes.

## Conclusion

The II/ID individuals, but not the DD group, presented PEH for SBP in both experimental exercise sessions. However, both groups had a protective effect of aerobic exercise on preventing the increase of DBP and MAP during post-exercise period. The endothelial responses of NO to exercise were only presented by the ID/II ACE genotype group and, similarly to PEH, seemed to be influenced by exercise intensity.

Therefore, the ACE genotype seems exert a role in the NO release and BP response during post-exercise recovery in elderly women. Any extrapolation of these results to other gender or age strata requires caution.

## List of Abbreviations

Angiotensin converting enzyme (ACE), nitric oxide (NO), blood pressure (BP), systemic arterial hypertension (SAH), post-exercise hypotension (PEH), insertion/deletion (I/D), angiotensin II (ANG II), renin-angiotensin system (RAS), nitrite (NO_2_^-^), resting before exercise (or control) in all testing days (PRE), immediately after exercise (IAE), blood lactate concentration ([Lac]), maximal incremental test (IT), rate of perceived exertion (RPE), ventilation (VE), oxygen uptake (VO_2_), carbon dioxide production (VCO_2_), ventilatory equivalent for oxygen (VE/VO_2_), dioxide carbon (VE/VCO_2_).

## Competing interests

The authors declare that they have no competing interests.

## Authors' contributions

HAPS, SRM, CBS, CSGC and HGS participated in the design of the study. HAPS, SRM, CBS, WBN and VNO performed the data collection. HAPS, SRM performed the statistical analysis. HAPS, SRM, MMS, RYA, FSE, OTN and HGS wrote the manuscript. All authors read and approved the final manuscript.

## Pre-publication history

The pre-publication history for this paper can be accessed here:

http://www.biomedcentral.com/1471-2261/11/71/prepub
